# Regulatory T Cells: Molecular Actions on Effector Cells in Immune Regulation

**DOI:** 10.1155/2016/1720827

**Published:** 2016-05-19

**Authors:** Asiel Arce-Sillas, Diana Denisse Álvarez-Luquín, Beatriz Tamaya-Domínguez, Sandra Gomez-Fuentes, Abel Trejo-García, Marlene Melo-Salas, Graciela Cárdenas, Juan Rodríguez-Ramírez, Laura Adalid-Peralta

**Affiliations:** ^1^Instituto Nacional de Neurología y Neurocirugía, 14269 México, DF, Mexico; ^2^Unidad Periférica del Instituto de Investigaciones Biomédicas en el Instituto Nacional de Neurología y Neurocirugía, 14269 México, DF, Mexico

## Abstract

T regulatory cells play a key role in the control of the immune response, both in health and during illness. While the mechanisms through which T regulatory cells exert their function have been extensively described, their molecular effects on effector cells have received little attention. Thus, this revision is aimed at summarizing our current knowledge on those regulation mechanisms on the target cells from a molecular perspective.

## 1. Introduction

T regulatory (Treg) cells are a T lymphocyte subpopulation that control the balance between immune activation and tolerance. Treg cells can originate from two main sources: thymus-generated natural Tregs (tTreg) and peripheral inducible Tregs (pTreg), generated during immune priming.

Several factors are required for tTreg generation; these cells are strongly dependent on TCR and CD28 signals and on several cytokines. Cytokines contribute to Treg maintaining via *γ* chain signaling of IL-2 and IL-15, and TGF*β* increases FOXP3 expression [1]. However, certain cytokines like as TNF-*α* have a controversial role in tTreg generation [2, 3].

On the other hand, pTreg generation requires stimulation in an anti-inflammatory milieu, a process where dendritic cells are critically involved [3, 4].

According to cytokine production, Tregs have been further classified; for instance, Th3 cells are characterized by TGF*β* production; Tr1 cells produce IL-10, and Tr35 cells produce IL-35. The suppressive capacity of these subsets is contact-independent. While the expression of the transcription factor FOXP3 can be transient in humans, this factor is associated with a suppressive function in mice. Thus, mouse Treg cells show the classical phenotype CD3+CD4+CD25+FOXP3+ [1].

## 2. Molecular Mechanisms of Treg-Mediated Suppression

Several action mechanisms by which Treg cells control the immune response have been reported: (1) inhibition by immunoregulatory cytokines such as TGF*β*, IL-10, and IL-35; (2) inhibition by cytolysis of effector cell by producing granzyme and perforin; (3) metabolic interruption, including an inhibition of the proliferative response via IL-2 receptor, cAMP-mediated metabolic inhibition, and immunomodulation mediated by the A2 adenosine receptor; (4) interaction with dendritic cells that modulates their function and maturation. A molecular description of each regulation mechanism is given below [3].

## 3. T Regulatory Cells Inhibit the Immune Response through Cytokine Production

### 3.1. Interleukin-10

IL-10 is an 18-kD protein produced by Tr1 cells. While its production is not restricted to this cell line since monocytes, dendritic cells, neutrophils, other T lymphocytes, and B lymphocytes are able to release it, Tr1 cells are the only regulatory cells to produce IL-10 [5, 6].

IL-10 has been described as the main immunomodulatory cytokine; additionally, it can act as a paracrine or autocrine signal and can be induced by catecholamines [7, 8].

IL-10 inhibits the production of inflammatory cytokines such as IL-12, causing a decrease in the Th1 response and in INF-*γ* production; it also promotes the phagocytic activity, increasing the removal of cellular debris at the inflammation site [9].

**Figure 1:**
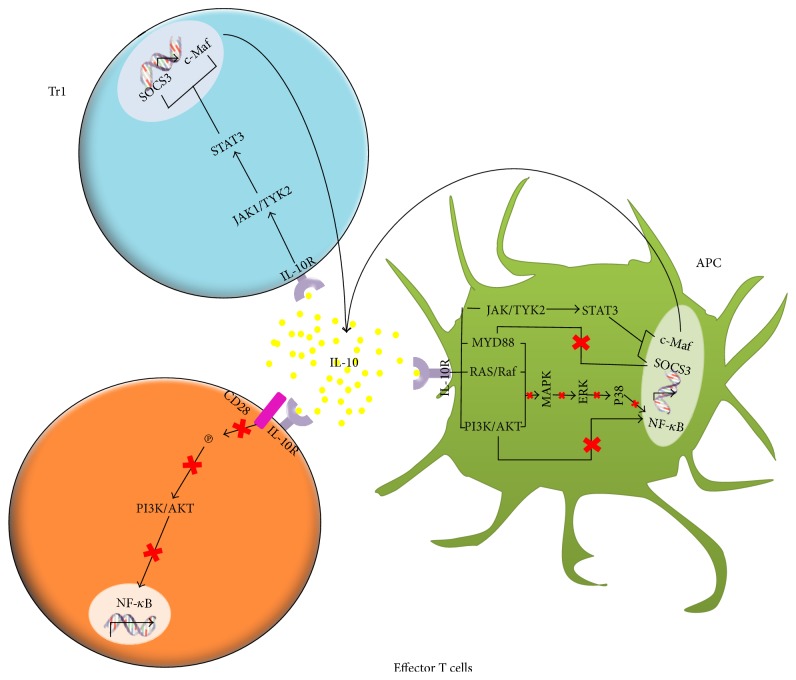
Interleukin 10 can act on effector cells, dendritic cells, and cytokine-producer T regulatory cells (Tr1). On effector cells, IL-10 exerts an immunosuppressor function. In dendritic cells, IL-10 can favor a tolerogenic phenotype that promotes the production of further IL-10. On Tr1 cells, IL-10 has an activator effect that also favors IL-10 production.

One of the best known molecular mechanisms of IL-10 is the action on effector cells. The costimulatory molecule CD28 is involved in the interaction between effector cells and antigen-presenting cells. By binding its receptor, IL-10 inhibits tyrosine phosphorylation in CD28, inhibiting PI3K/AKT activation, which in turn inhibits the signaling cascade leading to NF-*κ*B translocation (Figure 1) [10, 11].

As shown in Figure 1, IL-10 exerts its biologic function by activating JAK1 and TYK2, both proteins associated to STAT1, STAT3, and in certain cases STAT5 (Figure 1) [10, 12]. In rats, the activation of the IL-10 receptor in dendritic cells has been observed to promote the JAK1-TYK2-STAT3 pathway. When activated, this pathway inhibits dendritic cell maturation, decreasing the expression of MHC II, CD11b/c, CD80, and CD86 [13]. In monocytes, IL-10 has been demonstrated to induce the expression of the SOCS3 suppressor gene, which has influence on IFN-induced tyrosine phosphorylation in STAT1 [10]. On the other side, in a LPS-induced inflammation model, IL-4 has been observed to promote c-MAF expression in activated macrophages; c-MAF binds the promoter of the IL-10 gene, favoring its production (Figure 1) [14, 15].

By binding its receptor in Tr1, IL-10 induces JAK1 and TYK2 phosphorylation, activating STAT3, which is translocated to the nucleus, promoting SOCS3; in turn, SOCS3 inhibits the NF-*κ*B-induced factor MYD88, resulting in the inhibition of the IL-1*β*, IL-6, and TNF-*α* cytokines (Figure 1) [16]. Moreover, it has been observed that IL-10 promotes STAT3 phosphorylation and its translocation to the nucleus in Treg cells, mediating a further IL-10 production (Figure 1) [17].

IL-10 exhibits a wide range of biological activities, including immunosuppression, anti-inflammation, and immunomodulation. IL-10 is able to inhibit MHC I expression in B and T cells and also in dendritic cells, all of them involved in the inflammatory response [13].

### 3.2. TGF*β*

The Transforming Growth Factor-beta (TGF*β*) family includes several structurally and functionally related proteins acting as multifunctional factors in a wide range of biological processes. TGF*β* is a 25 kDa protein and a multifunctional cytokine, given its different effects on the cell [18, 19]. During secretion, TGF*β* undergoes proteolysis by an endopeptidase, which cleaves the peptide bond linking the mature factor and the Latency-Associated Peptide (LAP). For TGF*β* to be activated, it must be dissociated from LAP. This process can be triggered by a number of factors, like temperature and pH changes. Three TGF*β* isoforms are known in humans, 1, 2, and 3, each coded in a different chromosome [20].

Functional TGF*β* receptors are types I and II (TGF*β* RI and TGF*β* RII), and nonfunctional receptors include types III, IV, and V. TGF*β* RI and RII bind TGF*β*-1 and TGF*β*-3 with greater affinity than TGF*β*-2. All normal cells have surface receptors for TGF*β*-1. TGF*β* RI and TGF*β* RII are responsible for the biological effects of TGF*β*-1 in mammal cells. However, TGF*β* RIII is also capable of binding TGF*β*-1. While TGF*β* RIII has no role in signal transduction, it has been suggested that it controls the availability of TGF*β*-1 in the local extracellular microenvironment and regulates its active presentation to the functional receptors [21, 22].

#### 3.2.1. TGF*β* Transduction Pathways

**Figure 2:**
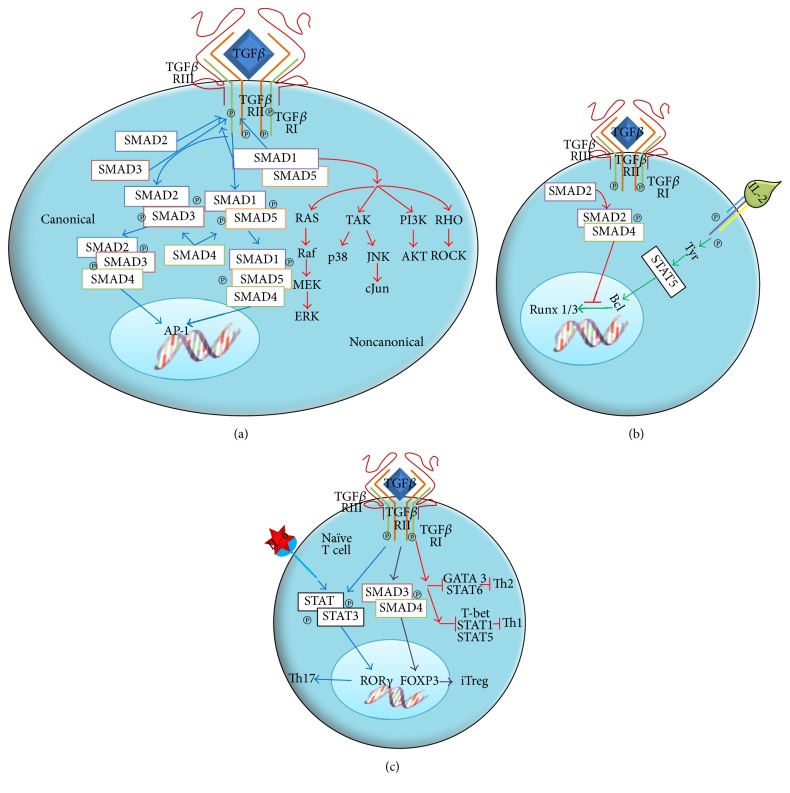
(a) The union of TGF*β* to its receptor activates both the canonical and noncanonical signaling pathways. (b) TGF*β* inhibits the proliferative response, either by inhibiting the production of the IL-2 growth factor or by controlling the expression of regulatory factors of the cell cycle. (c) TGF*β* has a key role in the differentiation of Th17 and Treg cells.

In the extracellular space, TGF*β* binds TGF*β* RIII, which then recruits TGF*β* RII and phosphorylates itself; alternatively, TGF*β* can bind directly to membrane-anchored TGF*β* RII and induce the attraction of TGF*β* RI and its ensuing phosphorylation (Figure 2(a)). Both TGF*β* RI and TGF*β* RII have a cysteine-rich extracellular region, a transmembrane region, and a cytoplasmic region; the latter includes a serine-threonine kinase domain. There is a functional dependence between TGF*β* RII and TGF*β* RI, since TGF*β* RI requires TGF*β* RII to bind the ligand, while TGF*β* RII requires TGF*β* RI for a functional signaling. When TGF*β* binds TGF*β* RII, the latter phosphorylates TGF*β* RI in the serine- and glycine-rich domain near the transmembrane region. The activation of the ligand-receptor complex allows the direct interaction of SMAD proteins with the kinase domain of the type-I receptor, recruiting SMAD2 and SMAD3 and phosphorylating them, thus activating the canonical pathway and forming a complex with SMAD4. This protein acts as a convergence node for the signaling pathways induced by members of the TGF*β* superfamily (Figure 2(a)). The active complex formed by SMAD2, SMAD3, and SMAD4 is translocated to the nucleus, where it acts as transcriptional coactivator and regulates the transcription of several TGF*β*-responder genes [21, 23].

TGF*β* activates TAK-1, a kinase of serine and threonine residues of the MAP kinase family, by the noncanonical pathway. RAS, a member of the small G proteins, has also been involved in signaling. Certain MAPKs, including those kinases regulated by extracellular signals such as ERK-1 and ERK-2 and stress-activated kinases such as JNK and PI3K, are also activated in some cell types (Figure 2(a)) [23].

TGF*β* is capable of regulating the effect of several immune cell types. A number of mechanisms have been proposed: (i) it suppresses effector T cell differentiation; (ii) it promotes the differentiation of naïve T cells into regulatory T or Th17 cells; (iii) it inhibits T and B cell proliferation; (iv) it inhibits the activity of macrophages, dendritic cells, and NK [23].

The inhibitory activity of TGF*β* on Treg cells is due to the high LAP/TGF*β* expression in Treg membrane. Unlike T cells, monocytes and dendritic cells express TGF*β* receptors, thus allowing for cell-to-cell interaction. Additionally, TGF*β* regulates dendritic cell maturation and differentiation. TGF*β* has been demonstrated as necessary to generate tolerogenic dendritic cells (DCs) by inducing IDO, an enzyme that inhibits T cell proliferation.

As another relevant suppressive mechanism, TGF*β* inhibits the production of IL-2 (Figure 2(b)). The cis-actin protein, which suppresses IL-2 production, is called TOB; this protein binds SMAD2, blocking the activation of RUNX 1/3. Additionally, it could interact with NFAT, a factor involved in IL-2 production. However, the SMAD2/NFAT interaction has not been proved yet. TGF*β* modulates cell proliferation by controlling the expression of cell cycle-regulating factors, including Cyclin-Dependent Kinases (CDKs) like p15, p21, and p27 and cell cycle promoters like cMYC, cyclin D2, CDK2, and cyclin E.

TGF*β* also promotes T naïve cell differentiation into Treg cells by inducing FOXP3 expression, or into Th17 cells in the presence of IL-6 (Figure 2(c)). Treg differentiation is dependent on TGF*β*, which activates SMAD3. SMAD3 enters the nucleus in association with SMAD4 and induces FOXP3 expression. FOXP3 then induces the transcription of inhibitory cytokines such as TGF*β* (Figure 2(c)). On the other hand, Th17 differentiation from naïve T cells is achieved by the combined action of TGF*β* and IL-6. Signaling via IL-6 activates STAT3, which induces the expression of ROR*γ*. ROR*γ* then induces the transcription of IL-17 (Figure 2(c)).

### 3.3. Interleukin 35

Interleukin 35 (IL-35) is a heterodimeric cytokine in the interleukin 12 (IL-12) family. This cytokine family can be composed of one to five subunits (p19, p28, p35, p40, and another from Epstein-Barr virus gene 3, also called EBI3). IL-35, an immunosuppressor cytokine formed by the p35 and EBI3 subunits, is produced by Treg cells [24]. IL-35 has two known functions: to suppress the proliferation of T helper cells and to promote the conversion of naïve T cells into highly suppressor Treg cells (iTr35) [25]. Recently, this cytokine was demonstrated to be capable of inducing the conversion of B lymphocytes into B regulatory cells [26].

The IL-35 receptor is formed by the IL-12R*β*2 and gp130 subunits; it can be heterodimeric or homodimeric. While gp130 is expressed in practically all cells, IL-12R*β*2 is expressed predominantly in activated T lymphocytes, NK cells, and to a lesser extent DCs and B cells [24].

The IL-35 signaling path has not been completely described yet; however, it is known that the heterodimeric receptor activates STAT1 and STAT4, which induces EBI3 and p30 expression [25], causing naïve T cells to convert into IL-35-producing (iTreg) cells, suppressing cell proliferation, blocking the shift to a Th1 profile, and mediating IL-10 production [27].

Treg cells are able to release the immunomodulatory cytokines IL-10, TGF*β*, and IL-35. This action may target several immune cells expressing receptors for such cytokines. This way, several immune cells in different stages (activated, effector, or resting) could be affected by the receptor-ligand interaction. The effect of cytokines is probably not only local, since they could spread by the bloodstream throughout the body. Thus, these cytokines play a role in the polarization of immune response during various pathologies.

## 4. Treg Cell Regulation Mechanism by Cytolysis of Effector Cells through Granzyme and Perforin

Regulatory T (Tregs) cells produce a serine protease called Granzyme B, which allows them to induce apoptosis in effector T cells [28–32].

**Figure 3:**
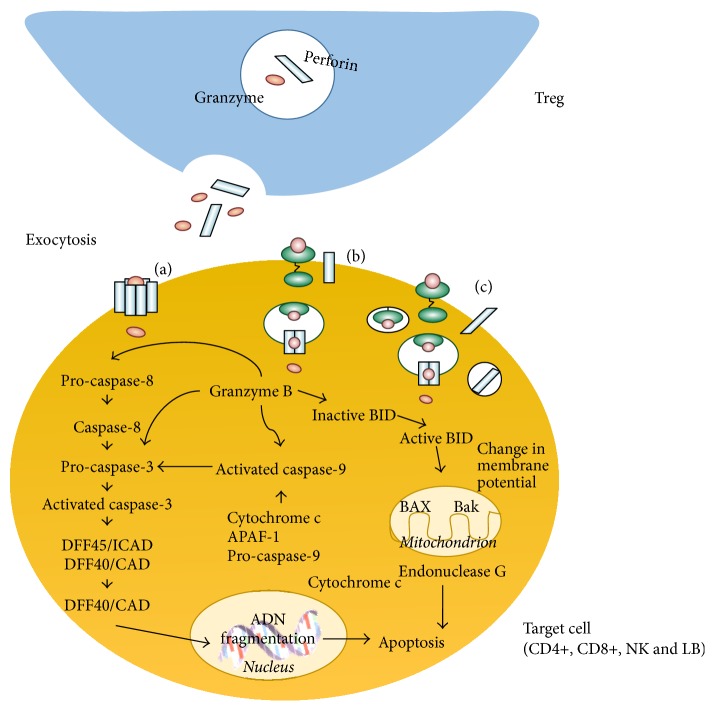
T regulatory cells can induce the death of effector cells by releasing granzyme and perforin. Granzyme can enter the effector cell through the perforin-mediated channels (a), or it could be endocyted through receptors (b) and perforin damage (c).

During Treg-effector cell interaction, directed exocytosis from Treg granules to the extracellular space of both cells takes place; these granules contain granzymes and perforins. Once released from the Treg, perforin molecules insert themselves into the lipid membrane of the target cell and polymerize in the presence of calcium ions to form a transmembranal cylinder; each cylinder forms a pore through which granzymes enter the cell (Figure 3(a)).

Granzyme can also enter the cell by an endocytosis process mediated by the manose-6-phosphate receptor. In this case, granzyme is sequestered in endosomes into the cytosol, and perforin acts to release it (Figure 3(b)) [33–37]. Additionally, granzymes can bind to the cell surface in a way that granzyme recruitment is stimulated by perforin-mediated membrane damage (Figure 3(c)).

Once within the target cell, Granzyme B could induce apoptosis by caspase-dependent or independent mechanisms, as discussed elsewhere [38–40].

Cytolysis allows Treg cells to act on several immune cell populations by cell-to-cell interaction. This mechanism is highly effective, since Treg cells induce death by apoptosis on effector cells, thus decreasing the number of effector cells and controlling the immune response.

## 5. T Regulatory Cells Inhibit the Immune Response by Metabolic Interruption

### 5.1. Inhibition of the Proliferative Response by Competition for IL-2

Interleukin 2 (IL-2), chiefly secreted by T cells in response to antigenic stimuli, is the main cytokine for T cell proliferation. The gene coding for this cytokine is located in chromosome 4 [41]. IL-2 receptor is expressed by T lymphocytes, NK cells, B cells, macrophages, and monocytes; however, only T lymphocytes are capable of producing this cytokine [42].

IL-2 receptor is a complex formed by three subunits: alpha chain (CD25), beta chain (CD122), and gamma chain (CD132); each subunit plays an important role in facilitating the transduction of IL-2-dependent signals [43]. Alpha chain (IL-2R*α*) has a very short cytoplasmic domain and does not participate in signal transduction; however, it is required to increase the affinity of IL-2 to its receptor. On the other hand, beta (IL-2R*β*) and gamma (IL-2R*γ*) chains play a crucial role in signal transduction [43].

**Figure 4:**
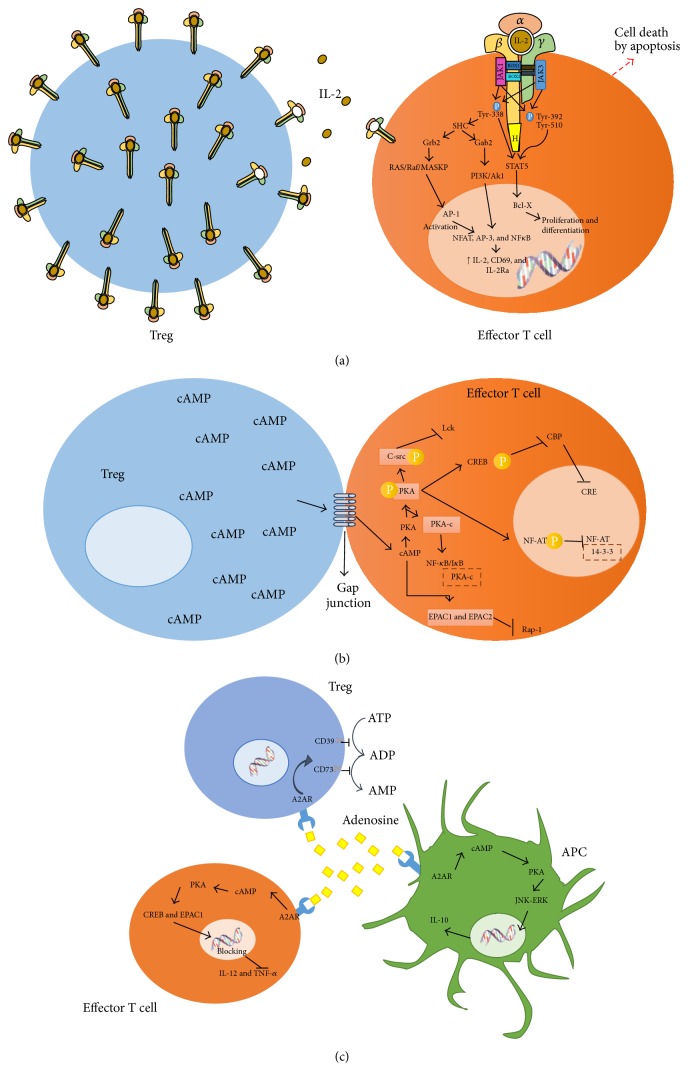
T regulatory cells can inhibit the immune response by three mechanisms: (a) By competition with effector cells for IL-2. (b) Through cAMP-mediated immunosuppression. (c) By adenosine production via the ectoenzymes CD39 and CD79.

Treg cells constitutively express high levels of IL-2 alpha chain, having thus a higher affinity to IL-2, and compete for this growth factor with proliferating cells. By depriving proliferating effector cells from IL-2, Treg cells do not only prevent them from continuing the proliferative process but also leave them without a vital cytokine, causing metabolic interruption and cell death (Figure 4(a)) [3, 43].

### 5.2. cAMP-Mediated Immunosuppression

Cyclic adenosine monophosphate (cAMP) is a second messenger, capable of regulating the functional activity of effector cells and antigen-presenting cells. The high cAMP content in Treg cells is due to the 50-fold higher expression of adenylyl cyclase 9 (AC9) [44]. Additionally, the high CD25 expression in Treg cells has been observed to favor adenylyl cyclase 7 activation and cAMP accumulation. The differential expression of CD25 and adenylyl cyclase in Treg cells is controlled by FOXP3. Tregs show a low cAMP degradation rate due to a diminished expression of the phosphodiesterase 3b enzyme in this cell subpopulation [45]. Recent findings indicate that cAMP concentration is controlled by miR-142-3p, a microRNA that regulates FOXP3 transcription activity, and thus the expression of adenylyl cyclase [46]. While miR-142-3p inhibits the production of adenylyl cyclase 9 in conventional T cells, this effect is not seen in Treg cells since the FOXP3 transcription factor negatively regulates miR-142-3p expression and keeps the AC9/cAMP pathway active [46].

Treg cells transfer cAMP to target cells by intercellular communications called gap junctions (Figure 4(b)). In mammal cells, communicating junctions are used for the bidirectional traffic of ions, metabolites, and other molecules weighing less than 1 kD. The increase in intracellular cAMP activates Protein Kinase A (PKA), since cAMP binds to the PKA regulating subunit, activating it by releasing its catalytic subunit. On the cell membrane inner face, phosphorylation of tyrosine kinase C-src (Csk) by PKA increases its activity; then, Csk phosphorylates and inactivates the lymphocyte-specific protein tyrosine kinase (Lck), an important protein in the proximal activation of T cell receptors. A number of signaling pathways can be regulated by PAK. For instance, the cAMP Response Element-Binding (CREB) protein is phosphorylated by PAK at serine 1343, which prevents it from forming a complex with the Csk Binding Protein (CBP) and from binding to cAMP Response Elements (CRE); these can be found in genes coding for T cell receptors or in other genes involved in T cell activation. Additionally, PAK regulates the activity of the nuclear factor in activated T cells (NF-AT). When NF-AT is phosphorylated by PAK, binding sites for another protein called 14-3-3 are created. This new complex decreases NF-AT transcription activity (Figure 4(b)) [47–49].

When cells are in resting state, NF-*κ*B is found in the cytoplasm, complexed with its inhibitor, I*κ*B, which prevents it from translocating to the nucleus. During cell activation, I*κ*B is phosphorylated by an I*κ*B kinase, which induces the NF-*κ*B/I*κ*B complex to split apart. Conversely, when PKA is phosphorylated, its catalytic subunit (PKA-c) binds to the NF-*κ*B/I*κ*B complex, stabilizing it and keeping it inactive (Figure 4(b)). NF-*κ*B regulates the transcription of a large number of genes, including those coding for the proinflammatory cytokines TNF-*α* IL-1b, IL-6, IL-8, VEGF, and metalloproteases (MMP1, MMP2, MMP3, and MMP13) [48].

While the main target for cAMP is PAK, cAMP has also been demonstrated to directly activate the exchange protein directly activated by cAMP (EPAC1 and EPAC2). This protein regulates the activation of a GTPase called Rap-1, responsible for activating ERK, thus inhibiting cell proliferation and differentiation (Figure 4(b)) [47].

### 5.3. Immunosuppression Mediated by the 2A Adenosine Receptor

CD39 and CD73 are ectoenzymes, highly expressed on the surface of Treg cells (Figure 4(c)). CD39 is a nucleoside triphosphate diphosphohydrolase-1 (NTPDase 1) that degrades ATP into AMP. The expression of CD39 in Tregs is regulated by the FOXP3 transcription factor, and its catalytic activity is enhanced by TCR compromise [50]. In turn, extracellular AMP is rapidly degraded into adenosine by CD73, an ecto-5′-nucleotidase bonded to the membrane of Treg cells (Figure 4(c)) [51].

The adenosine resulting from AMP hydrolysis binds four different surface receptor subtypes coupled to Gs proteins, called A1, A2A, A2B, and A3. The A2AR receptor is the main adenosine receptor associated to T and B lymphocytes, NK cells, macrophages, dendritic cells, and granulocytes (Figure 4(c)) [52].

The outcome of stimulating these receptors is an intracellular AMP accumulation; through the cAMP-dependent protein kinase, these signals phosphorylate and activate CREB. The latter binds the nuclear cofactor p300, producing a complex that regulates the expression of several genes in their promoter regions. CREB is able to regulate indirectly the transcription of some inflammatory genes, competing with NF-*κ*B/p65 and then suppressing the expression of proinflammatory cytokines like TNF-*α* (Figure 4(c)) [51, 53–55].

AMP can also activate other substrates like EPAC1 and other kinases such as ERK and JNK, altering the expression of proinflammatory genes through transcription factors responsible for synthesizing interleukins like IL-12 and TNF-*α*; it promotes as well the production of anti-inflammatory cytokines like IL-10 (Figure 4(c)) [56].

The fact that Tregs produce adenosine and respond to it at the same time means that this molecule acts as an autocrine factor to optimize the anti-inflammatory response. It also increases Treg suppressor capacity, inhibits the expression of costimulatory molecules in dendritic cells, and inhibits the activation of effector cells [52, 55].

Treg-mediated metabolic disruption occurs by competition for IL-2, a growth factor for effector cells. Under these conditions, Treg cells control the immune response in a nonspecific but effective manner, since by consuming IL-2 Treg cells inhibit effector cell proliferation, actually impairing the immune response. On the other hand, immunosuppression mediated by cAMP and the 2A adenosine receptor induce several signaling pathways in effector cells that in turn impact transcription factors, controlling the effector response.

## 6. Cell-Cell Interaction between Tregs and Dendritic Cells

### 6.1. Interaction through CTLA-4

Tregs can interact with DCs through CTLA-4. This molecule has a high affinity by dendritic cell-expressed CD80 and CD86; thus, Tregs compete with effector cells to bind these ligands.

**Figure 5:**
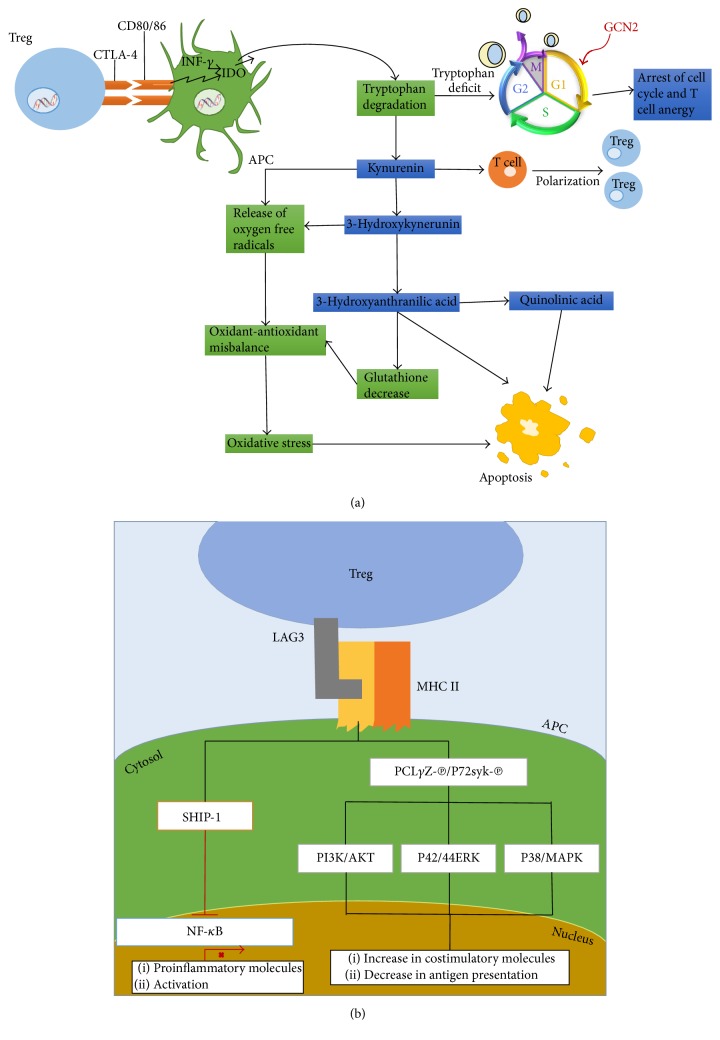
T regulatory cells have contact direct with dendritic cells through CTLA-4 (a) and LAG3 (b).

This interaction involves several events, including the production of INF-*γ*, a potent inducer of Indoleamine 2,3-dioxygenase (IDO). This enzyme catalyzes the conversion of tryptophan into an immunoregulator metabolite [57–59]. IDO degrades tryptophan in a metabolic pathway starting with tryptophan oxidation to N-formylkynurenine, immediately followed by hydrolysis to kynurenine. This initial product follows various degradation pathways whose final products are kynurenic acid, 3-hydroxykynurenine, anthranilic acid, 3-hydroxyanthranilic acid, and quinolinate (Figure 5(a)) [59–61]. Once tryptophan is degraded by IDO, a signaling cascade starts. Since there is a local tryptophan deficit, the level of transfer RNA without amino acid load increases, and as a result the General Control Nonderepressible Kinase 2 (GCN2) is activated, and it hyperphosphorylates the Translation Initiation Factor (eIF2), leading to a suppression in protein synthesis. The union of the ternary complex formed by eIF2, GTP, and the initiation transfer RNA for methionine (Met-RNAi) to the 40S ribosome subunit is inhibited, preventing the release of the binary complex eIF2/GTP and inhibiting the eIF2-promoted GTP/GDP exchange. The result is a cell cycle arrest in the G1 phase and thus anergy of effector T cells (Figure 5(a)) [60, 62, 63].

Additionally, IDO exerts an immunosuppressive activity through the metabolites resulting from tryptophan degradation. Although the mechanism is not well understood, 3-hydroxyanthranilic acid and quinolinic acid have been demonstrated to induce apoptosis in Th1 cells by directly activating caspase-8 [64, 65]; this truncates the BID protein, producing a small proapoptotic fragment called truncated BID (tBID). This active fragment can translocate to the mitochondria, causing the release of Cyt c by interaction with BAX, another member of Bcl-2 family. In the cytosol, Cyt c interacts with the proteins APAF1 and caspase-9 in the presence of ATP, building the apoptosome, a catalytic complex that starts the caspase cascade activation, leading to the digestion of structural proteins, degradation of chromosomal DNA, and phagocytosis of apoptotic bodies (Figure 5(a)) [66, 67].

The metabolite 3-hydroxyanthranilic acid has also been reported to induce death in activated T cells by depleting glutathione, one of the main antioxidant molecules in animal cells. A decrease in glutathione promotes a misbalance between ROS production and the antioxidant capacity [68]. On the other side, T cell apoptosis due to oxygen free radicals produced by kynurenine and 3-hydroxykynurenine has been observed, leading as well to changes in the oxidant-antioxidant balance and promoting oxidative stress. Oxidative stress causes a global collapse of mitochondrial function, reducing energy production and therefore contributing to cell death [64, 69]. Another immunosuppression mechanism involves kynurenine binding to the Aryl-hydrocarbon receptor (Ahr), a transcription factor expressed in cell populations like T cells, resulting in a shift to a T regulatory cell phenotype (Figure 5(a)) [70, 71].

### 6.2. Interaction through LAG3

Lymphocyte-activation gene 3 (LAG3, CD223) is a cell surface molecule expressed in Tregs [72]. Structurally, LAG3 is homologue to the CD4 receptor and binds to MHC II with a significantly higher affinity than CD4 [73–75].

The LAG3-MHC II union induces a signaling cascade starting with PLC*γ*2 and p72syk phosphorylation and the activation of PI3K/AKT, p42/44ERK, and p38MAPK (Figure 5(b)). The latter kinase has been involved in the maturation process of dendritic cells; these exhibit an increase in the expression of costimulatory molecules and a decrease in antigen capture [76, 77].

Another study demonstrated that, after crosslinking MHC in the presence of LAG3, ITAM-mediated inhibitory signals are induced, involving ERK sequential activation and SHIP-1 recruitment similar to those proposed for FC*γ*R*γ*. The inhibitory signaling started by coengagement of MHC with LAG3 along with CD4 and TCR could be mediated by several components, including SHIP-1, which may not be recruited by themselves by the CD4 or TCR signaling pathways. SHIP-1 has been labeled as a NF-*κ*B negative regulator and as a negative factor in cell activation [78]. Additionally, the conjoint union of CTLA-4 and LAG3 promotes a tolerogenic phenotype in DCs (Figure 5(b)) [77].

Dendritic cells being a key component of the immune response, the action implying cell-to-cell contact with DCs is one of the most important mechanisms for Treg cells. Depending on its phenotype, a DC can activate or control the immune response. When interacting with a Treg cell, a DC acquires a tolerogenic phenotype, which in turn promotes further Treg cell generation, providing a suppressor microenvironment. The competition for DC ligands between effector and Treg cells allows for an additional control mechanism of the immune response.

## 7. Conclusion

The action mechanisms of Tregs described above can act together or independently, according to the requirements of the immune system and homeostasis maintenance, or during the progression of various pathological processes. Treg cells have been used as part of an escape mechanism by several pathogens, including viruses [79] and helminthes [80], and they also modulate the immune response in noninfectious pathologies such as tumors. Knowing the modulation molecular mechanisms and their effect according the pathological situation could help in identifying the therapeutic targets allowing an effective immune response.
